# The blood-sucking tick *Ixodes hexagonus* reveals dietary stable isotope signatures of mammalian hosts

**DOI:** 10.1371/journal.pone.0327245

**Published:** 2025-07-03

**Authors:** Gaia G. Mortier, Stuart Black, Andrew C. Kitchener, Katherine A. Sainsbury, Robbie A. McDonald, Georg Hantke, M. Alejandra Perotti

**Affiliations:** 1 Department of Geography and Environmental Science, University of Reading, Whiteknights, Reading, England; 2 National Museums Scotland, Chambers Street, Edinburgh EH1 1JF, Scotland and School of Geosciences, University of Edinburgh, Drummond Street, Edinburgh, United Kingdom; 3 University of Alberta, Edmonton, Alberta, Canada; 4 University of Exeter, Penryn Campus, Penryn, England; 5 School of Biological Sciences, University of Reading, Whiteknights, Reading, England; Alaska Pacific University, UNITED STATES OF AMERICA

## Abstract

Ticks are obligate haematophagous (‘blood-sucking’) ectoparasites that are capable of retaining host dietary traces post-moult, providing an opportunity to investigate parasite–host interactions and explore their potential as non-invasive subsampling techniques. However, research on the preservation of biochemical host signatures within whole engorged parasites remains limited. Here, we examine stable isotope ratios of nitrogen (δ^15^N) and carbon (δ^13^C) across different tick tissues (exoskeleton vs. blood meal) and between whole ticks and one of their hosts, the European polecat *Mustela putorius*. Additionally, carbon and nitrogen weight percentages (wt%) are assessed to explore potential biochemical changes linked to blood meal digestion. Our findings showed that the isotopic composition of tick exoskeleton and blood meal differed significantly, with exoskeletons potentially reflecting a previous host. Whole engorged ticks showed a close δ^15^N relationship to their host, consistent with that of trophic enrichment, while the observed δ^13^C values were more variable. These findings enhance our understanding of how haematophagous parasites preserve host dietary signatures and, with further research, could support their use as a valuable alternative to invasive sampling methods, particularly when destructive sampling is not feasible.

## Introduction

Several biochemical methods, such as molecular and stable isotope analysis (SIA), have been utilised to establish parasite host species preference, as this forms an important element in the transmission cycles of vector-borne diseases [1–4]. Stable isotope analysis can assist in untangling complicated ecological dynamics, such as those found in parasite–host relationships, by tracking the flow of nutrients through ecosystems as they move from food source to consumer [5–11]. Yet, studies on the isotopic interactions between parasites and hosts, particularly those involving obligatory blood-sucking external parasites (‘ectoparasites’) such as ticks, remain relatively scarce. Ectoparasites have the potential to be useful biomonitoring tools and non-invasive host subsamples, as their exoskeleton incorporates the isotopic signature of (previous) hosts during moulting, and the blood meal allows direct analysis of the current host [12,13]. However, the understanding of how isotopic traces of host diet are reflected across parasite tissues remains limited.

As organic tissues grow (or ‘turn over’) at different rates, they incorporate isotopes over varying timespans [14,15]. Hair, whiskers, and muscle tissue turnover rates have been reported to reflect a dietary average ranging from one to three months across various mammals [16–19]. In contrast, blood is continuously synthesised and regenerates at a faster rate, allowing for a high-resolution reflection of diet up to the last 24 hours (plasma) or a couple of weeks (red blood cells) [14]. Arthropod exoskeletons are composed of proteins and chitin secreted by the epidermis [20]. Once newly formed exoskeleton hardens, it remains inert and does not grow until the next moult.

**Fig 1:**
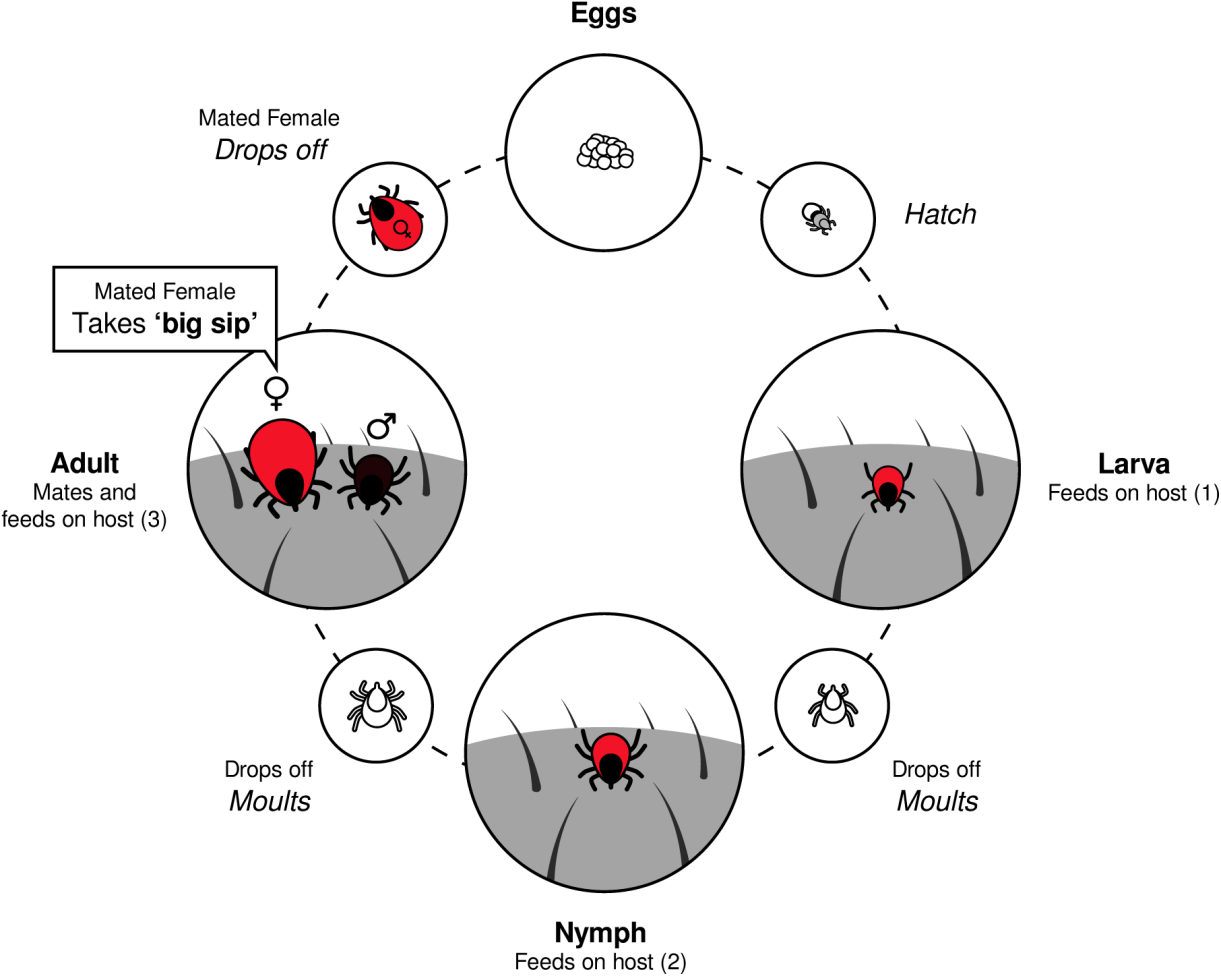
Typical life cycle of a three-host ixodid tick. Larvae hatch from eggs before finding a host and taking a blood meal, after which they drop off the host to moult into nymphs. Nymphs repeat this process, moulting into adults after feeding. On the host, males seek out females to mate with while taking small meals themselves and repeating this process several times before dying. Mated females will engorge themselves on blood to fuel their final act; dropping off their host and laying thousands of eggs [21].

Many tick species within the genus *Ixodes* (Acari: Ixodidae), including *Ixodes hexagonus*, follow a three-host cycle, feeding once each as a larva, nymph, and adult (Fig 1) [21,22]. Feeding durations vary, with nymphs and larvae feeding for up to five days, unmated females for up to 12 days, and mated females for up to 15 days before undergoing a final rapid engorgement stage (‘big sip’), increasing their weight by up to 35 times [20,23–27]. Digestion occurs continuously during the initial ‘slow feeding’ phase and resumes once the tick has detached, with larvae and nymphs sometimes taking months to fully process a single meal. The nutrients are then used to grow a new cuticle and moult, or in mated adult females, for egg production [20,28]. *Ixodes hexagonus* (Leach, 1815), commonly known as the hedgehog tick, is a nest-dwelling species primarily found on the European hedgehog *Erinaceus europaeus* [21,26,29,30]. Yet, it has been widely reported to feed on a small range of other medium-sized mammals, with European polecats *Mustela putorius* being particularly common hosts [28,31–35]. While polecats are generalist carnivorans, British polecats mainly feed on lagomorphs, such as the European rabbit *Oryctolagus cuniculus*, whereas in other parts of Europe, their diet varies more widely and is influenced by local prey availability [36–40].

Several studies have shown that freshly moulted ticks retain the isotopic composition of their larval host, enabling identification of the host they fed on during a previous life stage [5,41–45]. This also applies to mated female ticks, who transmit residual host biomarkers into their eggs and larvae [46]. Yet, the distribution of isotopes within engorged ticks and their potential as host diet proxies remains underexplored. The sampling of ectoparasites has potential applications in biomonitoring and host species identification, offering a minimally invasive method for blood sampling [47–52]. This is particularly valuable for studying endangered animals or in unique historical museum specimens, where destructive sampling of host tissues may not be an option. This study used isotopes commonly associated with dietary analysis, namely nitrogen (δ^15^N) and carbon (δ^13^C), to analyse the relationships between ticks and one of their hosts, the European polecat. Specifically, stable isotope ratios and biochemical composition were examined to assess differences between tick exoskeletons and blood meals, as well as between engorged ticks and their host.

## Materials and methods

### Study materials

From 2012–2016 the Vincent Wildlife Trust conducted a national polecat survey across Great Britain, collecting polecat carcasses primarily found as roadkill. Further examination was conducted at National Museums Scotland (S1 Table). During this analysis, ticks were removed from the polecats and identified to species using Arthur’s (1963) taxonomic key [53] before being transferred to 70% ethanol, which has been shown to have no significant effect on stable isotope preservation [54]. Ten European polecat hosts were sampled for both whisker (*n = *10) and muscle tissue (*n *= 6) where available, with three *I. hexagonus* ticks from each host (*n *= 30) undergoing isotopic analysis. All ticks used in this study were adult females that were visibly engorged. An additional set of ticks (*n *= 6) belonging to four of the polecat hosts was selected to undergo dissection. The coagulated blood and internal organs (‘blood meal’) were separated from exoskeletons through an incision in the ventral abdomen, using fine forceps and a scalpel under a stereomicroscope.

### Ethics statement

No live animals were used in this study. All polecat specimens were collected post-mortem by the Vincent Wildlife Trust as part of a national roadkill survey (2012–2016) and provided for scientific use. Ticks were removed during processing at National Museums Scotland. No fieldwork was conducted by the authors, and no permits or ethical approval were required for the use of these pre-collected materials. This research complies with all relevant institutional and national guidelines for the ethical use of animal specimens.

### Stable isotope analysis

Polecat muscle tissue was subsampled after being stored at −40°C since necropsy examination was completed in 2016. Lipids were extracted from polecat muscle and whiskers using a 2:1 v:v chloroform:methanol solution whilst sonicating for 10 minutes and rinsing with ultra-high-quality water (UHQ, 17 MΩ) in between, until the waste solution ran clear. The samples were dried at 40°C for 48 hours, after which muscle tissue underwent a 48-hour freeze-drying process. The freeze-dried muscle tissue was homogenised using mortar and pestle. Polecat whiskers were sampled in small sequential sections to account for temporal variations in isotopic composition. Whole ticks, tick blood meals, and tick exoskeletons were rinsed thoroughly using UHQ water before undergoing freeze-drying for 48 hours under vacuum, to remove moisture and preserve them in a dry state suitable for isotope analysis. Tick exoskeletons were subsampled from the abdomen using a scalpel, whereas blood meals and whole ticks were crushed using mortar and pestle. Samples were weighed in triplicate (0.15–0.25 mg), using a Sartorius Cubis MSA6.6S-000-DM microbalance and enclosed within a 6 x 4 mm tin capsule. The stable isotope ratios of carbon and nitrogen were measured using a continuous flow-isotope ratio mass spectrometer coupled with a ThermoFisher™ DeltaV Advantage fitted with an Isolink CNSOH Temperature Conversion Elemental Analyzer (TC/EA) and smart function at the Chemical Analysis Facility at the University of Reading. The findings are presented as δ values per mil (‰) relative to the global benchmarks of Vienna PeeDee Belemnite (VPDB) for carbon, and atmospheric N_2_ for nitrogen. The resulting data was adjusted for linearity and instrument drift per 5 samples, as well as stretch corrected using in-house (x3) and international standards (x5, IAEA CH-7, USGS 56, 62, and 63). Comparisons between measured values and expected values were conducted to determine analytical error, which was < 0.15 ‰ for δ13C and δ15N.

### Statistical analyses

All statistical analyses were performed using Minitab 21.0.0. Tick and European polecat δ^15^N and δ^13^C values were tested for normality and homogeneity of variances using Anderson-Darling and Levene’s tests. If data did not meet the assumptions, δ^15^N and δ^13^C values were transformed (log_10_ + 30 and log_10_, respectively). In order to establish the isotopic offset between tick exoskeleton and blood meal, discrimination factors (Δ^15^N or Δ^13^C) were calculated as the difference between the isotopic ratios (δ^15^N or δ^13^C) of tick tissues using the following equation: Δ^15^N or Δ^13^C = δ^X^_exoskeleton_ – δ^X^_bloodmeal_, where δ^X^_exoskeleton_ and δ^X^_bloodmeal_ represent the δ^13^C or δ^15^N values of the tick exoskeleton and blood meal belonging to the same tick. To investigate potential relationships between carbon wt% and nitrogen wt% in tick blood meals, a Pearson correlation coefficient was used, and the nitrogen-to-carbon ratio (C:N = N wt%/ C wt%) standardised to atomic mass (14/12) was considered. To account for multiple ticks from the same host, a mixed-effects model was fitted with type (tick or host) and tissue (tick blood meal, tick exoskeleton, whole tick, host muscle, host whisker) as fixed factors, with δ^15^N and δ^13^C values as variables. Host ID was included as a random factor. To assess the preservation of the host’s chemical fingerprint within ticks, discrimination factors (δ^15^N and δ^13^C) were calculated between whole ticks and their host tissues (whisker and muscle). As whiskers represent incremental periods of isotopic incorporation, the isotopic values associated with the most recent growth period were used. A linear regression analysis was performed to model the relationship between host and parasite tissue types as predictors, with δ^15^N and δ^13^C values acting as response variables. Significance was assumed at α = 0.05 in all cases.

## Results

**Fig 2:**
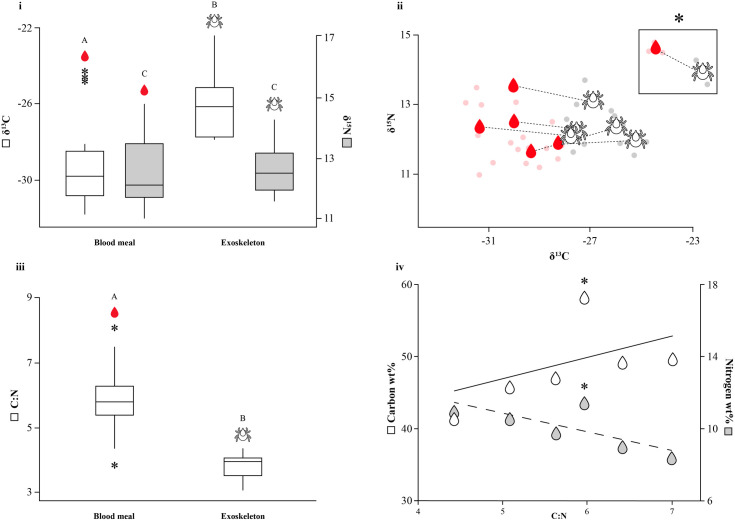
Nitrogen and carbon isotope ratios (‰) in tick blood meals and exoskeletons. Mean (i) δ^13^C (in white) and δ^15^N (in grey) in ‰, (ii) stable isotope ratios of nitrogen (δ^15^N) and carbon (δ^13^C), (iii) mean carbon to nitrogen ratio (C:N) values of tick blood meals (*n *= 6) and exoskeletons (*n *= 6). (iv) carbon wt% and nitrogen wt% of blood meals with regression lines, where each pair of data points represents an individual tick. Tukey grouping shows statistical significance, dotted lines signify samples belonging to the same tick, and outliers are indicated by *.

The stable isotope ratios of carbon (δ^13^C) and nitrogen (δ^15^N) were measured in tick blood meal (*n* = 6) and exoskeleton (*n *= 6) from four polecat hosts. The δ^13^C values of tick blood meals were found to be significantly more negative (^13^C-depleted) than those of exoskeletons (LMM: −1.45 ± 0.089, p < 0.001; Fig 2i, ii; S2 Table), whereas no significant difference was found for δ^15^N values (−0.001 ± 0.119, p = 0.993). Tick blood meals (−0.09 ± 0.117, p = 0.441 for δ^15^N; −0.108 ± 0.057, p = 0.071 for δ^13^C) and exoskeletons (−0.19 ± 0.117, p = 0.117 for δ^15^N; 0.039 ± 0.061, p = 0.525 for δ^13^C) showed a lower intra-triplicate variance in δ^15^N (but not δ^13^C for exoskeletons) when compared to whole ticks. However, these differences were not statistically significant. The C:N ratios of whole ticks (4.95 ± 0.67; Fig 2iii) were closer to those of blood meals (5.94 ± 0.89) than exoskeletons (3.92 ± 0.3). The relationship between nitrogen wt% and carbon wt% levels within blood meals revealed a weak negative correlation (Pearson: r = −0.066; Fig 2iv), potentially indicative of digestion effects. When statistical outliers belonging to a single tick were excluded, the negative correlation became much stronger (Pearson: r = −0.843).

**Fig 3:**
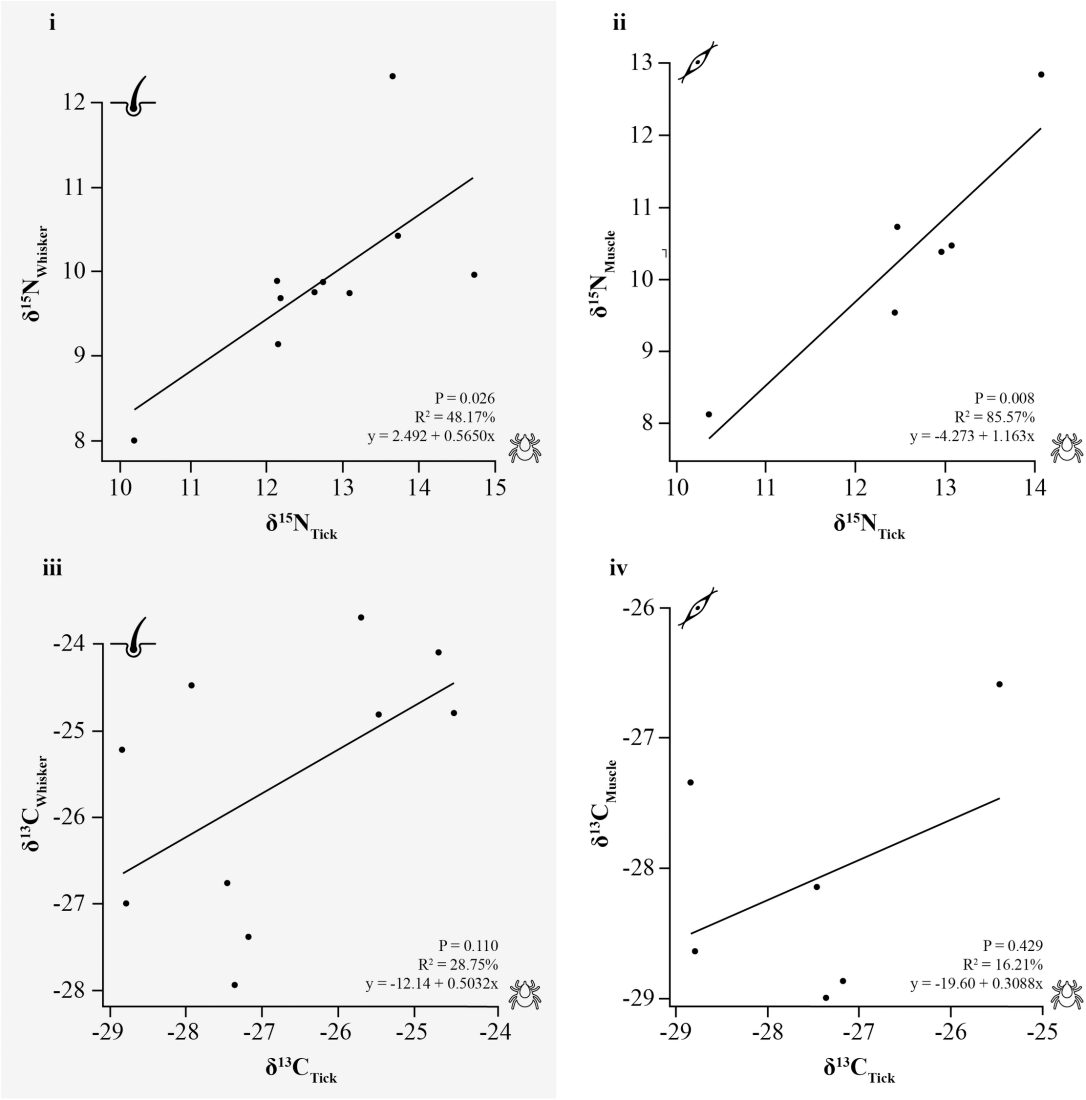
Regression lines of nitrogen and carbon isotope ratios (‰) between ticks and polecat host tissues. Relationships between δ^15^N (i-ii) and δ^13^C (iii-iv) in ‰ of (i, iii) polecat whisker and whole tick, and (ii, iv) polecat muscle and whole tick, including regression lines.

Furthermore, the stable isotope ratios in whole engorged ticks (*n* = 30) recovered from ten European polecat hosts (whiskers and muscle) revealed that ticks showed significantly higher δ^15^N values compared to those of their hosts (LMM: −1.389 ± 0.154, p < 0.001 and −0.44 ± 0.171, p = 0.011, for whisker and muscle respectively; S3 Table). The observed δ^13^C values showed more variability in both polecats and ticks. Whole tick values were significantly more negative when compared to those of host whiskers (1.214 ± 0.158, p < 0.001), but significantly less negative in comparison to those of host muscle tissue (−0.948 ± 0.175, p < 0.001). Tick isotope values were observed to mirror the patterns seen across host individuals. Linear regression analyses between whole ticks and polecat whiskers revealed a moderate positive linear relationship for δ¹⁵N (R² = 0.482; p = 0.026), but less so for δ¹³C (R² = 0.287; p = 0.110; Fig 3). Similarly, a significant positive linear relationship was found between muscle tissue and whole ticks for δ¹⁵N (R² = 0.856; p = 0.008), but not for δ¹³C (R² = 0.162; p = 0.429).

## Discussion

Our study found a significant difference in δ^13^C between tick exoskeletons and the blood meals preserved within them. This supports the theory that exoskeletons retain isotopic traces from a prior host, whereas blood meals are indicative of the current host; though it is important to consider that the observed offset may be a result of an inherent isotopic difference between tissue types. In contrast, the reported δ^15^N values for tick exoskeletons and blood meals do not strongly separate host sources. This may reflect the lower individual-level variability in δ15N among polecats, as observed in the consistent nitrogen isotope values of both muscle and whisker tissues. Previous studies established that ticks retain their hosts’ isotopic signature even after moulting, but these findings were based on ticks that had fully digested their last blood meal before moulting [5,43,44]. As the samples used in this study were collected from a natural environment, the isotopic composition of the ticks’ exoskeletons reflect previous feeds, and thus, an unidentified previous host. The period between nymphal and adult life stages in ticks can range from months to years in temperate regions, as some species rely on specific seasonal conditions to locate their preferred hosts [55]. In generalist ticks, this previous host is more likely to be a completely different species, whereas in the nest-dwelling *I*. *hexagonus*, repeated feeding on the same host species cannot be ruled out [23,31,33]. In contrast, the blood meal is almost certainly from the individual polecat the tick was recovered from during collection. Our findings indicate greater intra-sample variation in whole ticks, likely due to their heterogeneous composition, where exoskeleton and blood meal components contribute distinct isotopic signals. The observed trend in nitrogen wt% and carbon wt% of tick blood meals from the same polecat host likely reflects different digestion stages, as nitrogen is lost more rapidly during digestion while carbon is metabolised more gradually to conserve energy [44]. This could furthermore explain the observed negative values in the lipid-rich blood meals, as lipids tend to be more ^13^C-depleted. In ixodid ticks, digestion and waste elimination occur during the slow feeding phase and after detachment, with nitrogenous waste rapidly excreted as guanine, while carbon is released more gradually as carbon dioxide through respiration [22,27,56,57]. However, mated female adult ticks enter an additional ‘big sip’ stage after the slow feeding process, during which they halt digestion altogether [22]. Therefore, it is during this specific life stage that ticks are most likely to contain undigested host blood, which could serve as a biochemical proxy.

**Fig 4:**
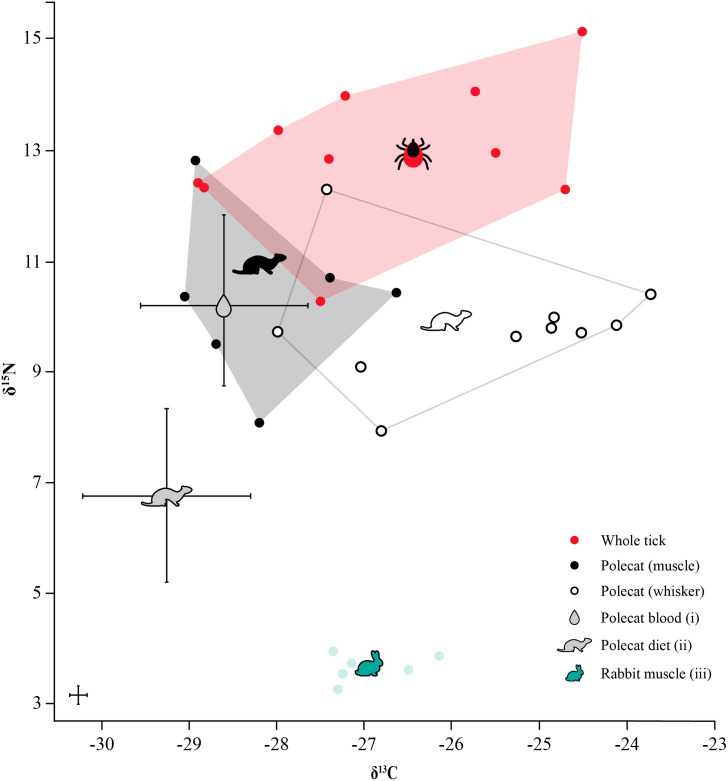
Nitrogen and carbon isotope ratios (‰) of ticks and their host, the European polecat (whiskers and muscle), as well as rabbit muscle, and polecat diet. Mean δ^15^N and δ^13^C (±SD in ‰) of whole ticks (each data point represents the mean of 3 ticks per host; *n *= 30), and polecat whiskers (*n *= 10) and muscle (*n *= 6). Polecat whole blood (i) and diet (ii) were calculated using muscle and data from red foxes *V. vulpes* [59]. Isotopic data (iii) from Neilson *et al.* [63] on British rabbit muscle (*n *= 6) were included as potential polecat prey. Analytical uncertainty is shown in bottom left.

This study further aimed to assess whether whole engorged ticks are capable of reflecting the dietary traces of their host through their isotopic signatures. Our results indicate that ticks showed a significant δ^15^N offset relative to their host, which is consistent with trophic enrichment and similar to those found in predator-prey relationships (Δ^15^N of around 3 ‰). A wide range of δ^13^C values was observed in both whole engorged ticks and polecats, and more so in whisker than muscle tissue (Fig 4). This variation may be due to several factors, including the diverse diet of the host, physiological processes within the ticks (such as digestion and excretion), temporal differences between the tick and polecat tissues, and potential post-mortem effects, considering the specimens may have been left to deteriorate several hours after death and prior to collection. The reported parasite–host offsets must be interpreted with caution due to limited sample size and host tissues analysed, as these did not contain the blood upon which the ticks directly fed. Several studies have found that mammalian blood isotopic values are overall similar in δ15N and more negative in δ13C compared to those of their fur and muscle tissue, aligning with our tick blood meal findings [58–60]. Using our polecat muscle data and the discrimination factors reported for the red fox Vulpes vulpes (Δ15NMuscle-Blood: −0.12 ‰ ± 1.13; Δ13CMuscle-Blood: −0.45 ‰ ± 0.07), as no polecat data are available, we estimated whole blood values (calculated as 55% plasma and 45% red blood cells), revealing that polecat blood δ^15^N and δ^13^C values were lower than those of tick blood meals (Fig 4) [59,61]. To better interpret the trophic position of ticks and assess their potential as host diet proxies, it is necessary to estimate the diet of their host. Currently, no tissue-to-diet offsets have been published for European polecats. Instead, the trophic discrimination factor for another generalist, the red fox, is commonly used in studies on medium-sized carnivorans (Δ15NMuscle-Diet = −3.5 ‰ and Δ13CMuscle-Diet = −1.1‰) [59,62].

When red fox discrimination factors were used to calculate diet from muscle, the isotopic values fell well below the carbon values reported for British rabbit muscle tissue, while their nitrogen values were higher [63]. This mismatch may reflect the dietary diversity of polecats. Although British polecats are known to mostly eat lagomorphs and other small mammals such as rodents, they are also known to feed on birds, amphibians, fishes, and reptiles [42]. Their lifestyle as a generalist carnivoran allows them to display great flexibility when it comes to prey selection based on availability, as seen in polecat populations across mainland Europe [36–39]. Assuming that polecat whiskers represent an average isotopic signature over roughly a month, while their muscle tissue reflects a period of several months or an entire season, the consistency in stable isotope values within these tissues indicates that the polecats maintained a consistent diet across these timescales. However, this does not imply that their range of prey is uniform, but rather signifies that polecats are consuming a similar range of prey over time [64]. Regression analysis revealed a significant positive linear relationship between nitrogen isotope values of host tissues and whole ticks, suggesting that characterising the polecat’s dietary patterns through their ticks is a possibility. Although this has promising implications for host diet reconstructions using stable isotopes and ectoparasites, several limitations are important to consider. For example, distinguishing between hosts with similar diets can be challenging, as previous studies have found that stable isotopes alone often fail to differentiate species with overlapping isotopic niches [5,41,43,45]. Despite their widespread use in food web reconstructions, carbon isotope values are often not taken into consideration when analysing parasite–host relationships, due to their inherent variability, which can be affected by, among other things, physiological processes and environmental factors [6,45**]**. However, δ^15^N has been reported as more reliable than δ^13^C for distinguishing hosts, with a combination of C:N ratios and δ^15^N providing the best distinction between host feeding treatments through parasite analysis [43,45].

In conclusion, the isotopic differences observed between tick exoskeletons and blood meals may reflect distinct hosts. This highlights the importance of separating these tissues when isotopically analysing engorged ticks, as mixing isotopic signals may reduce the reliability of interpretations. This could be addressed through blood meal dissection, or potentially through a compound-specific isotope approach. While the reported δ^15^N values suggested that ticks could act as isotopic proxies to hosts, the lack of a significant δ^13^C relationship indicated that additional data on carbon isotope fractionation in ectoparasites are needed. In addition, further research should investigate how tick digestion rates influence isotopic host signature, preferably in controlled feeding environments. The stable isotope analysis of ectoparasites remains an underutilised method for studying host-parasite interactions and could provide a useful alternative for reconstructing host diets, particularly in cases where direct tissue sampling is impractical.

## Supporting information

S1 Table Specimen metadata for European polecats (*Mustela putorius*) used in the study. This table lists accession numbers, species, collection years, and institutional details for museum specimens held at National Museums Scotland. These specimens were the source of *Ixodes hexagonus* ticks analysed in this study.
(XLSX)

S2 Table Summary of host and tick isotopic data. Includes δ^15^N and δ^13^C values (‰), elemental weight percentages, C:N ratios, and calculated discrimination factors between tick blood meal and exoskeleton tissues.
(XLSX)

S3 Table Stable isotope composition of polecat (*Mustela putorius*) whiskers, muscle, and associated whole ticks (*Ixodes hexagonus*). Includes δ^15^N and δ^13^C values (‰), nitrogen and carbon wt%, and atomic C:N ratios. Where applicable, values represent monthly whisker segments and averages of replicate tick samples.
(XLSX)
